# Supplementary material for: Genetic and Transcriptome Analysis of Leaf Trichome Development in Chinese Cabbage (Brassica rapa L. subsp. pekinensis) and Molecular Marker Development
Source: Int J Mol Sci. 2022 Oct 22;23(21):12721. doi: 10.3390/ijms232112721 (PMC9659260; doi:10.3390/ijms232112721)
Supplement: Supplementary file 1 [file ijms-23-12721-s001.zip › Supplementary figure.pptx]

## Slide 1
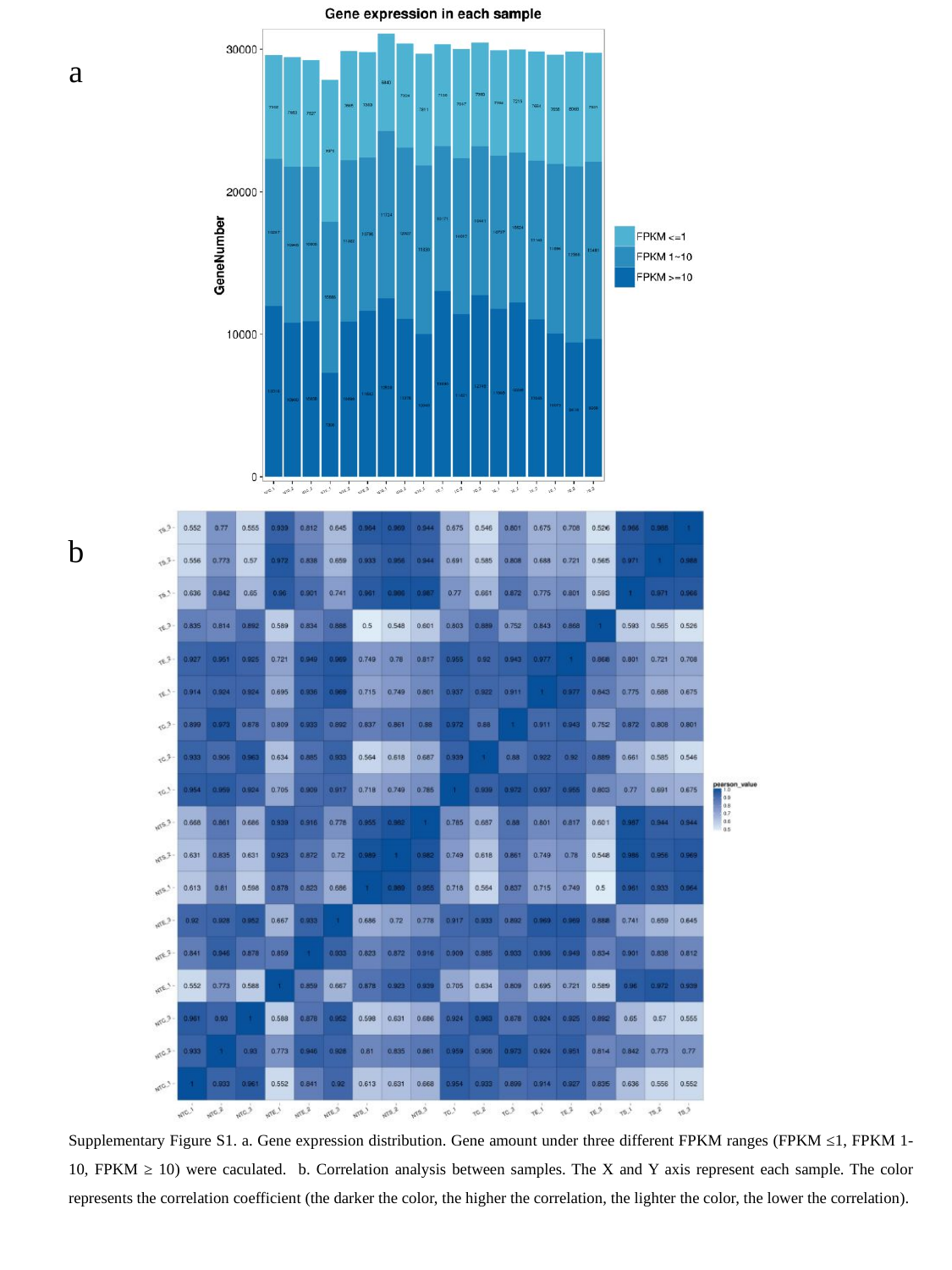

a
b
Supplementary Figure S1. a. Gene expression distribution. Gene amount under three different FPKM ranges (FPKM ≤1, FPKM 1-10, FPKM ≥ 10) were caculated. b. Correlation analysis between samples. The X and Y axis represent each sample. The color represents the correlation coefficient (the darker the color, the higher the correlation, the lighter the color, the lower the correlation).
